# Accessory Inferior Sulci of the Liver in an Afro-Caribbean Population

**Published:** 2016-06

**Authors:** Shamir O. Cawich, Michael T. Gardner, Ramnanand Shetty, Neil W. Pearce, Vijay Naraynsingh

**Affiliations:** 1 Department of Basic Medical Sciences, University of the West Indies, Mona Campus, Kingston 7, Jamaica;; 2 Department of Clinical Surgical Sciences, University of the West Indies, St. Augustine Campus, Jamaica

**Keywords:** Liver, Variation, Sulcus, Surface, Anatomy

## Abstract

**Introduction:**

There have been no previous reports on the anatomic variations that exist on inferior surface of the liver in Caribbean populations. This information is important to optimize radiology and hepatobiliary surgical services in the region.

**Methods:**

Two investigators independently observed 69 cadaveric dissections over five years and described the variations in surface anatomy.

**Results:**

In this population 88% of cadaveric livers had conventional hepatic surface anatomy. However, 12% had accessory sulci present on the visceral surface of the liver, with a 7:1 male preponderance. When present, there was 100% correlation between the presence of Rouvière’s sulcus and the right branch of portal pedicle.

**Conclusion:**

Abnormal surface anatomy is present in 12% of unselected specimens in this Caribbean population. Interventional radiologists and hepatobiliary surgeons practicing in the Caribbean must be cognizant of these differences in order to minimize morbidity during invasive procedures.

## INTRODUCTION

In classic descriptions of liver surface anatomy, the visceral (inferior) surface contains three named fissures and two fossae that define surface anatomy. The term accessory inferior sulcus (AIS) refers to the presence of extra fissures on the visceral surface of the liver. There has been no prior report on the prevalence of AIS in Caribbean populations, although it is well established that there are unique variations in this population (1, 2).

This study was carried out to document AIS prevalence in the Caribbean population. Knowledge of these variations is important to clinicians who treat liver disorders in persons of Caribbean extract.

## METHODS

Cadaver dissections were performed to facilitate anatomical teaching for post-graduate surgical residents at the University of the West Indies. Two independent investigators observed all consecutive cadaveric dissections at this facility over a period of five years. Upon opening the abdomen, the visceral liver surface was inspected in situ. We defined two groups of cadaveric livers: those with classic anatomy (3) and those with AIS present. Cadaveric livers with AIS were selected for detailed evaluation.

**Figure 1:**
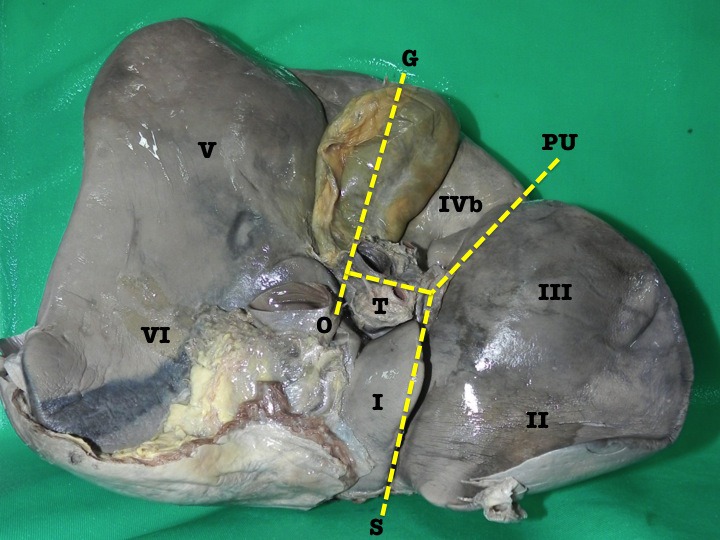
A view of the inferior surface of an explanted cadaveric liver demonstrating classic anatomy. The inferior surface is roughly divided into four lobes by the gallbladder fossa (G), transverse fissure (T), oblique fissure (O), saggital fissure (S) and porto-umbilical fissure (PU). Roman numerals denote the hepatic segments as described by Couinaud.

Figure 1 demonstrates the classic anatomy of the visceral liver surface divided into four lobes roughly in the shape of the letter “H” by the transverse fissure, gallbladder fossa, fossa for inferior vena cava (IVC), saggital fissure and the porto-umbilical fissure (3-6).

The porta hepatis (also known as the transverse fissure (4)) is a deep fissure that is approximately 5 cm in length (6). It extends transversely on the undersurface of the liver, separating segment I posteriorly from segment IV anteriorly (5). The hepatic portal triad enters the liver at the porta hepatis where the oblique, saggital and porto-umbilical fissures meet (4).

The oblique fissure (also known as the median fissure (5)) lies along Cantlie’s plane and extends from the gallbladder fossa to the IVC. The saggital fissure (also known as the fissure for ligamentum venosum (6) or left fissure (5)) forms the left border of the caudate lobe (segment I), separating it from segment II as it extends anteriorly to the porta hepatis. The porto-umbilical fissure (also known as the fissure for ligamentum teres (5)) forms the left border of the quadrate lobe (segment IV) and separates segments III and IV as it extends anteriorly to the liver edge.

Any additional sulci observed on the visceral surface of the liver apart from those aforementioned in the classic descriptions (3-6) were considered AIS. When they were observed, we documented the in-situ relationships of the AIS to nearby organs, including the kidney, colon, stomach and spleen. The livers were then explanted by interrupting the triangular and coronary ligaments, transecting the supra-duodenal hepato-duodenal ligament and transecting the IVC at least 2 cm above the insertion of hepatic veins and 2 cm below the lower border of the liver.

Each specimen was observed on the dissection bench. The number, location, depth and length of AIS were recorded. Measurements were taken using electronic calipers (General Tools, MFg Co., New York, USA) by two independent investigators. The average measurement was used as the final dimensions. The parenchyma was then dissected to document the course of hepatic veins, portal veins and hepatic arteries in relation to the accessory sulci. Finally, all specimens were sectioned in 1 cm saggital slices to detect the presence of parenchymal pathologies.

## RESULTS

Over the five-year study period, there were 39 male cadavers and 30 female cadavers dissected. A total of 8 (12%) cadavers (7 males and 1 female) had AIS present. The mean age of cadavers with AIS was 68 years (range 55-85).

The 7:1 male preponderance observed in the study cohort was statistically significant with a two-tailed P Value of 0.0101 on Fisher’s exact test. Interestingly, the AIS were always present on the right side of the liver and they were remarkably constant in their position. Two distinct sulci were observed (Table 1).

**Table 1:** Accessory Inferior Hepatic Sulci in an Afro-Caribbean Population

Case	Number of sulci	Location (segment)	Length (cm)	Depth (cm)	Special observations

1	1	Segments V/VI lying in a coronal plane extending from the transverse fissure	4.0	1.5	Open-type segment V fissure
2	1	Segments V/VI lying in a coronal plane extending from the transverse fissure	2.0	1	Hypertrophic left lateral segment
					Open-type segment V fissure
3	1	Segments V/VI lying in a coronal plane extending from the transverse fissure	2.0	2	Macro-nodular cirrhosis
					Open-type segment V fissure
4	2	Segments V/VI lying in a coronal plane extending from the transverse fissure	3.0	2	Closed-type segment V fissure
		Segment VI	2.0	1.5	
5	2	Segments V/VI lying in a coronal plane extending from the transverse fissure	2.5	1.5	Closed-type segment V fissure
		Segment VI	2.0	1.5	
6	2	Segments V/VI lying in a coronal plane extending from the transverse fissure	2.5	1.5	Closed-type segment V fissure
		Segment VI	4.0	2	
7	2	Segments V/VI lying in a coronal plane extending from the transverse fissure	2.0	1.5	Closed-type segment V fissure
		Segment VI	1.5	1	
8	1	Segments V/VI lying in a coronal plane extending from the transverse fissure	5.29	1.6	Closed-type segment V fissure

**Figure 2:**
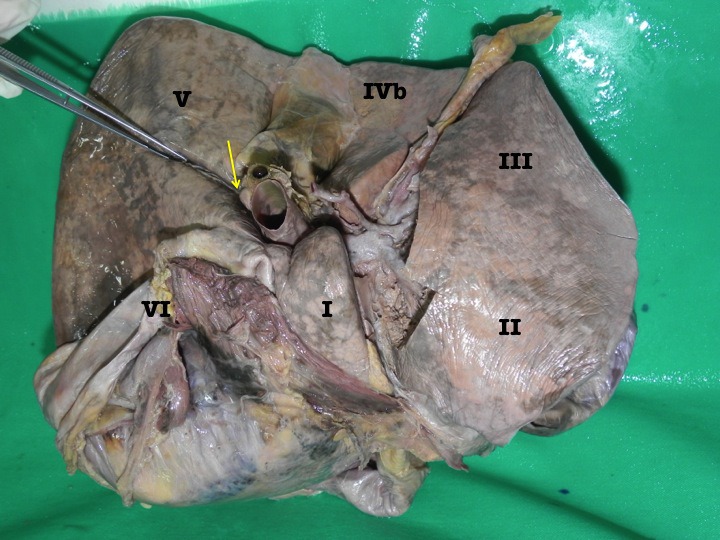
Photograph of the inferior surface of an explanted cadaveric liver in anatomic position. Couinaud’s segments are labeled for orientation. In this case, the accessory inferior hepatic sulcus extends continuously from the lateral end of the transverse fissure (yellow arrow) and extends into the right liver as demonstrated by the dissecting forceps.

In all cases, there was a deep sulcus observed in the right liver lying in a coronal plane, illustrated in Figure 2. It commenced medially at the lateral extent of the transverse fissure, often encroaching on the gallbladder neck. The sulcus extended postero-laterally for variable distances into the right liver, often touching the upper pole of the right kidney. The mean length of this fissure was 2.9 cm. This sulcus was usually well developed and covered with an invagination of Glisson’s capsule for a mean depth of 1.6 cm.

**Figure 3:**
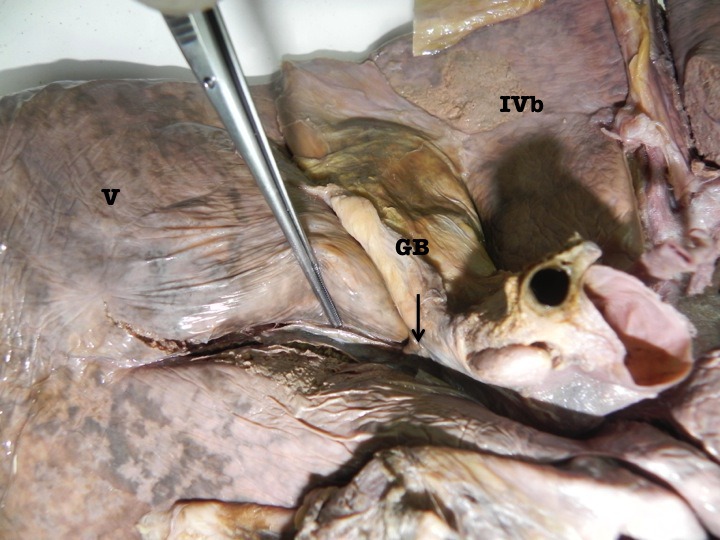
Photograph of the inferior surface of an explanted cadaveric liver in anatomic position. Couinaud’s segments are labeled in roman numerals for orientation. The accessory inferior sulcus is seen extending from the lateral edge of the transverse fissure (arrow) near the neck of the gallbladder (GB). The dissecting forceps points to the right branch of the portal vein seen after dissection of the parenchyma at the floor of the fissure.

**Figure 4:**
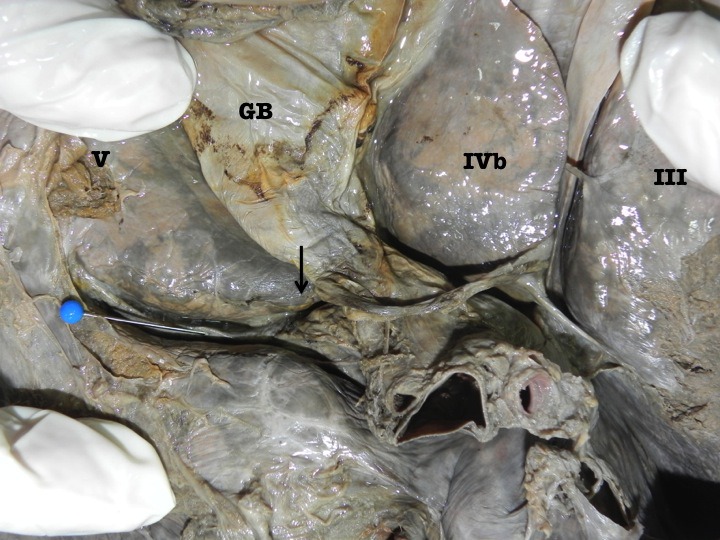
Photograph of the inferior surface of an explanted cadaveric liver in anatomic position. Couinaud’s segments are labeled in roman numerals for orientation. The accessory inferior sulcus is seen extending from the lateral edge of the transverse fissure (arrow) near the neck of the gallbladder (GB). The blue pin points to the right branch of the portal vein that lies at the floor of the fissure. In this case, there is no parenchymal bridge covering the right branch of portal vein.

When parenchymal dissection was carried out at the floor, it revealed that the sulcus bore a relationship to the right branch of the portal vein in all cases. The right branch of portal vein followed the sulcus for varying distances. In 5 cases, the right branch of portal vein was covered by a thin band of parenchyma at the floor of the AIS (Figure 3) and in the remaining 3 cases the right branch of portal vein could be seen at the floor of the sulcus without being covered by a parenchymal bridge (Figure 4).

**Figure 5:**
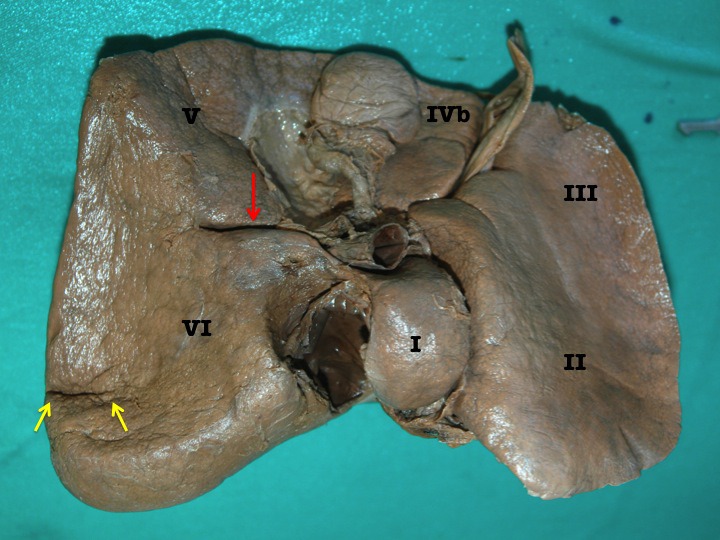
Inferior surface of an explanted cadaveric liver. Couinaud’s segments are labeled with Roman numerals. This specimen demonstrates both accessory inferior hepatic sulci. The red arrow indicates the consistent sulcus continuous with the transverse fissure. The yellow arrows point to the accessory fissure at segment VI.

In four of the specimens there was a well-defined AIS at segment VI (Figure 5). When present, it was well developed with a mean depth of 1.5 cm and a mean length of 2.4 cm. On in-situ examination, there were no abnormalities at adjacent viscera in any of the specimens. The colic, renal, gastric and duodenal impressions appeared normal. This sulcus bore no apparent relationship to any venous structures on parenchymal dissection.

**Figure 6:**
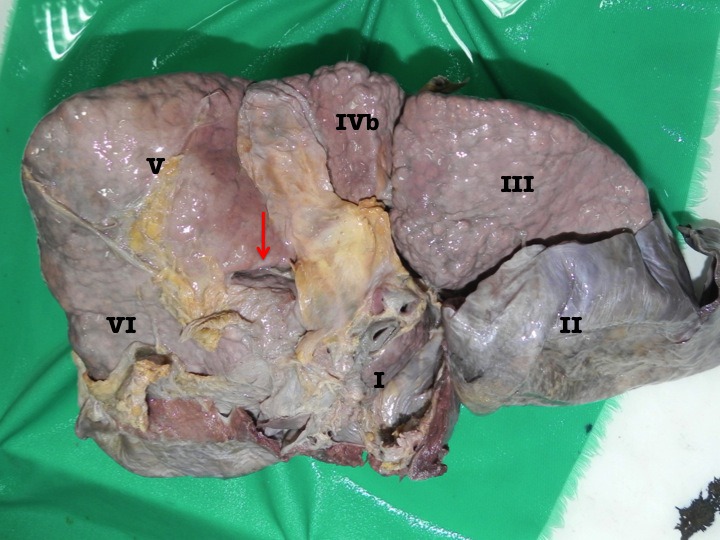
Explanted liver of index case 3. The architecture of this liver is preserved despite obvious macro-nodular cirrhosis. There is still a well-developed accessory sulcus (arrow) in segment V that is continuous with the porta hepatis and extending into the right liver. In this specimen, the right branch of portal vein can be seen at the floor of the sulcus without a covering parenchymal bridge.

**Figure 7:**
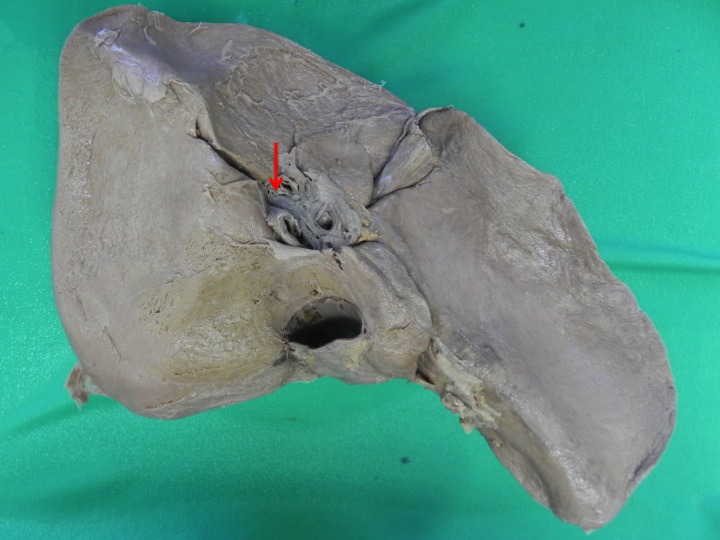
Explanted cadaveric liver of index case 2 with a hypertrophic left lateral segment. Despite the hypertrophy, the classic arrangement of hepatic sulci is present except for the consistent accessory inferior sulcus at segment V/VI (arrow).

We had no information on the cause of death or any information about pre-morbid diseases for these cadavers. However, the cadaveric livers appeared grossly normal on sectioning in 6 cases. Two cadavers had notable pathologic abnormalities. The liver of the cadaver labeled index case 3 had macro-nodular cirrhosis (Figure 6) and that from the cadaver labelled index case 2 had a markedly hypertrophic left lateral section but no other gross pathologic process (Figure 7). None of the specimens had intra-parenchymal liver pathologies detected on sectioning.

## DISCUSSION

Most existing reports document the presence of AIS in populations largely of Aisan (7, 8), Caucasian (9-14) and Indian descent (15). This is the first report of a series of AIS in an Afro-Caribbean population. There were notable differences in our study population when compared to the published reports from predominantly Caucasian (9-14) or Aisan (7, 8) populations. Macchi *et al*. (9) reported that accessory liver sulci were commoner in in females but there was a statistically significant male preponderance (7:1) in our population.

The first AIS we observed was a consistent and well-developed sulcus at the right liver commencing at the lateral extent of the transverse fissure near the gallbladder neck and traveling obliquely for 2.9 cm (mean) into the antero-lateral right liver. It closely matched the description of a sulcus originally described by Henri Rouvière in 1924 (10). In his original description, Rouviere described “le sillon du processus caude” as a cleft running to the right of the hilum anterior to the caudate process (10). Several names have since been used to describe this sulcus: Reynaud et al. ascribed the name “Incisura Dextra of Gans” in 1991 (11) and Lim *et al*. (7) termed it the “inferior accessory hepatic fissure” in 1986.

The prevalence of this sulcus ranges widely in the published literature (8, 12-15). The incidence ranges from as low as 0.8% in South Korea (7) to as high as 82% in Slovenia (12). In our Afro-Caribbean population the prevalence (12%) was significantly lower than reports from caucasian populations (9-14), but it was comparable to that reported by Muktyaz *et al*. (15) who found these sulci in 11% of cadavers in North India.

The average dimensions of Rouvière’s sulcus in our study were 2.9 cm in length and 1.6 cm in depth. This was comparable to the existing reports in medical literature (11-15). Similar to most existing reports, the sulci in our series were covered by an invagination of Glisson’s capsule, suggesting that they were true fissures (4, 7).

When Lim *et al.* (7) evaluated ultrasounds in 2000 unselected persons they noted that the sulcus extended from the right side of the porta hepatis at the level of the right branch of the portal vein and followed the proximal part of the posterior branch of the right branch of the portal vein. Several subsequent investigators have documented a relationship between the sulci and the right branch of portal vein using contrast enhanced CT scans (4, 8, 16-18). Dahmane *et al*. (12) reported that branches of the right posterior sectional pedicle were found in the Rouvière’s sulcus in 70% of the cases. Joshi *et al.* (8) and Lim *et al*. (7) both reported finding the right branch of portal vein at the depth of the sulcus in 100% of their cases.

We had similar observations in our setting where the right branch of portal vein was located at the floor of this sulcus in all cases. In our study the right branch of portal vein was visible at the floor of the sulcus without dissection in 38% of cases. Zubair *et al*. (14) described this variant as an open-type sulcus when the branches of the right hepatic pedicle were visible and the sulcus was continuous throughout its length. When Dahmane *et al*. (12) studied 33 cadaveric livers with Rouviere’s sulci, they reported that 85% were open-type sulci and the remaining 15% had a bridge of parenchyma covering the vessels. This was markedly greater than was seen in our population, where only 38% were the open-type variants.

Rouviere’s sulcus is important because it is used a landmark during laparoscopic cholecystectomy in order to reduce bile duct injuries (12, 14, 19, 20) since the cystic duct and artery are found anteriorly (14) while the common bile duct is found posteriorly (19). It is also an important landmark for hepatobiliary surgeons to identify the biliary pedicle to segments V and VI for tailored liver resections (12). In our population, its presence had 100% correlation with the presence of the right branch of portal vein.

We observed a second AIS that was relatively consistent in location at segment VI running in a coronal plane. Othman *et al*. (17) were the first to describe a transverse fissure posteriorly in segment VI “near the colic impression”. Their paper contains photographs of the sulcus that bear a remarkable similarity to the consistent segment VI fissure that was present in 6% of our cadavers. They suggested that “pressure exerted by the colon” was responsible for the segment VI fissure (17). However, the colon is relatively mobile and contacts a large surface area of the liver. Therefore, it seems unlikely that it would produce a single consistent fissure as a result of external pressure. Nevertheless, we could not find any obvious relations between gross anatomic abnormalities and this fissure on in-situ examination.

These sulci are believed to be true sulci that form because of peritoneal in-folding peritoneum during embryogenesis (17, 18, 21). They are believed to be different from furrows on the anterior surface of the liver that are believed to be false depressions due to pressure from overlying diaphragmatic slips (4, 22) or ribs (17, 23). Due to the observational nature of this study we were not able to determine the exact cause of the AIS. However, evidence does exist from animal models that support the theory of variations from events occurring during embryogenesis.

Suksaweang *et al.* (18) established the relationship between events during embryogenesis and resultant mass and shape of the liver by exposing chick embryos to various compounds regulating the activity of beta-catenin. Using this model, they demonstrated that beta-catenin expression stimulated hepatocyte proliferation and increased liver size (18). They proposed that growth could still occur after embryogensis at specific areas in the liver known as localized growth zones (18). Therefore, any stimulus that regulated beta-catenin could result in a change in the liver size, shape and the formation of accessory lobes.

Glinka *et al* (24) demonstrated the opposite effect when beta-catenin expression was suppressed by administration of inhibitory substances. Further evidence was provided later in experiments with mice by inhibition (25) and stimulation (26) of beta-catenin. Currently, the prevailing theory is that this is the mechanism through which the liver regenerates after hepatic resections (25). Harada *et al.* (27) also suggested that this could even lead to neoplastic disease after they demonstrated the ability of beta-catenin expressed from an adenovirus causing hepatomegaly in mice liver.

Johnson *et al.* (1) suggested that ethnicity could play a role in morphologic liver anomalies in the Afro-Caribbean population but we cannot substantiate these theories of genetic predisposition to anatomic variance without further research.

Knowledge of the prevalence and patterns of AIS is important information to any clinician treating liver diseases. It is important to radiologists and oncologists for proper image interpretation. Auh *et al.* (4) reported that only 1 in 4 AIS were correctly identified on CT scans and often they were mistaken for pathological lesions (8, 28). There have also been reports of mistaken diagnoses of hepatic abscesses and haematomas when fluid collects within these fissures (28). They are also important to hepatobiliary surgeons because they may impact the complexity of liver resections and transplantation (29, 30). These variations have become important in light of the establishment of and rapid progress in hepatobiliary services in the Caribbean in the past decade (31, 32).

The major limitation of this study is that it is a small observational study. In this study 69 cadaveric dissections were performed over five years. It may have been possible to detect other differences if larger numbers of cadaveric dissections were performed. However, it would have been difficult to accrue larger numbers because of the nature of the study and acquisition of cadavers for study.

## CONCLUSION

The prevalence of accessory inferior sulci differs from that reported in international literature. Rouviere’s sulcus is only present in 12% of persons in the Afro-Caribbean population, but when it is present there is 100% correlation with the right branch of portal vein. This is important information to any clinician treating liver diseases in persons of Caribbean extract.

## CONLICTS OF INTEREST

The authors declare that no conflicting interests exist.
